# How Configural Is the Configural Superiority Effect? A Neuroimaging Investigation of Emergent Features in Visual Cortex

**DOI:** 10.3389/fpsyg.2017.00032

**Published:** 2017-01-23

**Authors:** Olivia M. Fox, Assaf Harel, Kevin B. Bennett

**Affiliations:** Department of Psychology, Wright State UniversityDayton, OH, USA

**Keywords:** vision, perception, perceptual organization, emergent features, configural, fMRI, visual cortex, ventral visual pathway

## Abstract

The perception of a visual stimulus is dependent not only upon local features, but also on the arrangement of those features. When stimulus features are perceptually well organized (e.g., symmetric or parallel), a global configuration with a high degree of salience emerges from the interactions between these features, often referred to as emergent features. Emergent features can be demonstrated in the Configural Superiority Effect (CSE): presenting a stimulus within an organized context relative to its presentation in a disarranged one results in better performance. Prior neuroimaging work on the perception of emergent features regards the CSE as an “all or none” phenomenon, focusing on the contrast between configural and non-configural stimuli. However, it is still not clear how emergent features are processed between these two endpoints. The current study examined the extent to which behavioral and neuroimaging markers of emergent features are responsive to the degree of configurality in visual displays. Subjects were tasked with reporting the anomalous quadrant in a visual search task while being scanned. Degree of configurality was manipulated by incrementally varying the rotational angle of low-level features within the stimulus arrays. Behaviorally, we observed faster response times with increasing levels of configurality. These behavioral changes were accompanied by increases in response magnitude across multiple visual areas in occipito-temporal cortex, primarily early visual cortex and object-selective cortex. Our findings suggest that the neural correlates of emergent features can be observed even in response to stimuli that are not fully configural, and demonstrate that configural information is already present at early stages of the visual hierarchy.

## Introduction

One of the more well-known ideas to emerge from Gestalt psychology is that the whole is different than the sum of its parts (Koffka, 1935; Wagemans et al., 2012). A closely related phenomenon has been referred to as emergent features: the subjective perception of a visual stimulus is dependent not only upon the local features but also on the joint co-occurrence and arrangement of those features (e.g., Pomerantz et al., 1977). When the stimulus features are perceptually well-organized (i.e., form a cohesive structure) a global configuration with a high degree of perceptual salience emerges from the interactions between local features. When these emergent features correspond to task demands behavioral performance improves dramatically (e.g., Pomerantz, 1986). Emergent features have been found to play a key role in visual display design (Bennett and Flach, 1992), as they provide powerful tools for decision-making by effectively leveraging the natural perceptual skills of human observers (Woods, 1991). If a display has been designed successfully the salient emergent features will represent meaningful properties and relationships within complex work domains, a notion critical in the ecological approach to display design (e.g., Bennett and Flach, 2011).

The perception of emergent features is often regarded as an “all or none” phenomenon, present only when the local features are strongly grouped to produce salient higher order global properties (Pomerantz and Portillo, 2011). The majority of work on emergent features largely focuses on stimuli that are either configural (i.e., producing salient emergent features) or non-configural (not producing emergent features). Configural stimuli are comprised of local features intentionally arranged by the experimenter to produce the emergence of a global percept (i.e., an emergent feature). For example, many of the studies use experimental stimuli that are arranged to form either bilateral symmetry or parallelism (e.g., Pomerantz and Garner, 1973; Pomerantz and Schwaitzberg, 1975; Pomerantz et al., 1977). And while the identity of the local features are the same for both configural and non-configural stimuli, those of non-configural stimuli have been intentionally arranged to be void of such emerging properties, or arranged to form an extreme case of poor configuration (Pomerantz et al., 1977; Kubilius et al., 2011). In other words, configural stimuli are arranged to produce high-level, salient properties emerging from the interactions of the elements in the stimulus, whereas the non-configural stimuli are designed to be void of such emergent features (Pomerantz et al., 1977).

Critically, however, this dichotomous approach to emergent features overlooks the possibility that stimuli between these ends of the continuum may vary in their degree of configurality. Thus, a possibility that has not explicitly been considered is that emergent features vary in a quantitative rather than qualitative manner. This would imply that configurality might be achieved even in conditions that do not entirely satisfy the geometric properties characteristic of emergent features, for example, in configurations that approximate but are not in themselves symmetric and parallel orientations (for a similar argument in the face perception literature, see Schwaninger and Mast, 2005). Stated alternatively, will observers be capable of tolerating some degree of imperfection in the arrangement of the local features, and if so, to what extent? Accordingly, will they still retain perceptual access to the emergent features that aid task performance?

The current neuroimaging study aims to address these questions by examining how far perceptual stimuli can stray from what is usually considered a prototypical configuration, before their emergent features break down. Such a breakdown may be reflected as a decrease in behavioral performance, as well as a decrease in the magnitude of neural activity associated with configural processing. To investigate this possibility we adapted the stimuli set used to demonstrate *the configural superiority effect* (CSE, Pomerantz et al., 1977). A set of stimuli were developed that continuously and parametrically varied the deviation of their constituent features from symmetric and parallel orientations. We chose to focus on symmetry and parallelism, as they are two prime examples of emergent features often discussed in the context of the CSE (e.g., Pomerantz and Garner, 1973; Pomerantz et al., 1977; Mersch, 2014; Pomerantz and Cragin, 2015). This allowed us to explore the extent to which emergent features are continuously perceived, and correspondingly, how behavioral outcomes (response times, accuracy) are impacted by degrees of configurality (e.g., continuous degradation or a dichotomous break?).

We further used neuroimaging to help us determine whether the neural correlates of CSE are sensitive to deviations from the prototypical configurations often used in perceptual organization research. We measured the neural responses to the above set of stimuli, focusing on regions along the ventral visual pathway that have been previously shown to support configural processing (e.g., Chechlacz et al., 2015; Ward and Chun, 2015). This enabled us to determine the extent to which the CSE is manifest along the visual hierarchy; specifically, we investigated whether CSE reflects activation across multiple levels of the visual hierarchy (e.g., Altmann et al., 2003; Ban et al., 2006; Chechlacz et al., 2015), or whether it is supported by specific high-level areas in occipito-temporal cortex (OTC) (e.g., Lerner et al., 2001; Kubilius et al., 2011).

While early visual areas (EVA; e.g., V1, V2, V4) are known to be primarily responsible for processing simple local features, these regions have also been implicated in global shape processing, suggesting a more varied and complex role for early visual cortex (EVC) than traditionally thought (e.g., Kourtzi et al., 2003). Neuroimaging work focused specifically on the perception of global shapes emerging from local elements arranged at various orientations shows both EVA and higher level visual areas are involved in the processing of emergent features (e.g., Altmann et al., 2003). In contrast, other works have shown configural effects primarily in higher areas in the visual hierarchy such as object-selective lateral occipital complex (LOC: Malach et al., 1995), and were not able to demonstrate configural effects in EVC. For example, Kubilius et al. (2011) found a CSE in LOC in the form of a higher decoding accuracy to whole stimuli relative to parts, whereas in EVC decoding accuracy was higher to parts than to wholes).

In the present study, we utilize knowledge of the functional properties of visual areas in order to infer how emergent features are processed by examining both behavioral performance and neural activity in pre-defined regions of interest (ROIs). We ask whether configural effects will be observed across a range of “non-typical” configural stimuli and whether these effects can be seen only in higher-level, or in both high-level and lower-level areas of the visual hierarchy.

## Materials and Methods

### Participants

Eight Wright State University students were recruited (6 female, age range: 18–29, *M* = 24.5). All participants had normal or normal-corrected vision, normal color perception, the ability to read and write in English, and the ability to complete a MRI safely. All participants signed an informed written consent according to the institutional review boards of Wright State University and the Air Force Research Laboratory (AFRL) and Miami Valley Hospital (MVH) Human Investigation and Research Committee (HIRC), and received due compensation for their participation.

### Stimuli

**FIGURE 1:**
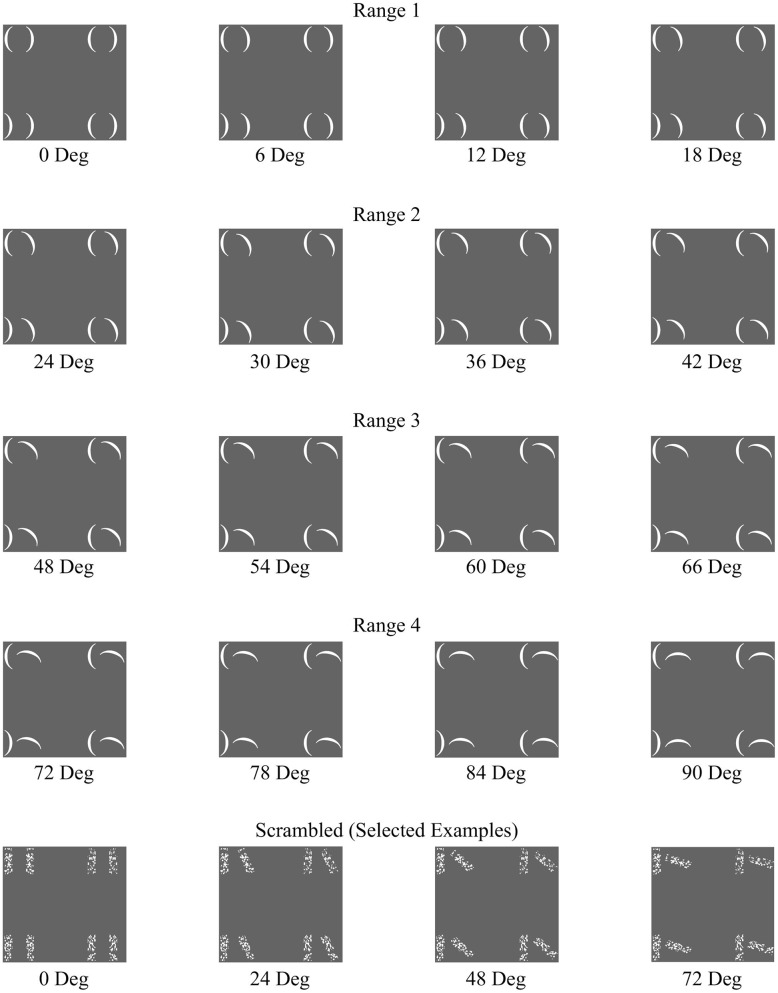
**A representative set of the full range of configural stimuli separated by Configurality Range, with a selected set of Scrambled stimuli**.

The stimuli comprised of 144 unique stimulus arrays. 128 of these stimulus arrays consisted of eight parentheses arranged to create four pairs aligned in a 2 × 2 matrix (**Figure 1**). Each pair subtended a visual angle of approximately 1.56° vertically and 1.79° horizontally. The overall array subtends a visual angle of approximately 7.15° vertically by 7.63° horizontally. The orientation angle of the rightmost parenthesis of each pair was manipulated to result in stimuli at 16 different rotational angles beginning with zero degrees rotation and continuing in six-degree increments (0°, 6°, 12°, 18°, 24°, 30°, 36°, 42°, 48°, 54°, 60°, 66°, 72°, 78°, 84°, and 90°). The target location and target direction were also manipulated such that on any given trial, the target could appear in any of the four quadrants, and be facing either left or right. This culminated in 128 unique stimulus arrays (16 angles × 4 quadrant locations × 2 target directions). In addition, 16 control stimulus arrays were used, which retained the 2 × 2 matrix structure, but using textural rectangular elements rather than the parentheses (**Figure 1**). Specifically, these “scrambled” arrays were made up of one parenthesis pair per quadrant with the rectangular area surrounding each parenthesis (2.1 mm × 7.0 mm) having been divided into 120 squares (0.35 mm× 0.35 mm) and then randomly rearranged while retaining each square’s original orientation. There were 16 versions of the Scrambled condition, one with the rightmost parenthesis of each pair oriented to each of the 16 rotational angles. In the Scrambled condition, there was no target.

### fMRI Procedure and Experimental Design

An event-related fMRI design was used for the study, with participants performing an anomalous quadrant discrimination task on the stimulus arrays. Stimuli were presented in a pseudo-randomized fashion, so that each sequence of 18 trials contained all 16 rotational angles stimuli, with an additional Scrambled stimulus array, and a null event (blank screen). The target location and target facing direction randomly varied across the sequences of stimuli, spanning the full range of the stimulus set. The stimuli were presented on the screen until a response was made (participants were instructed to maintain fixation throughout the length of the experiment). Immediately following the participant’s response a fixation cross screen of variable duration was presented (duration of the interstimulus interval was determined by multiplying the latency of the response time by two and adding a randomly chosen value ranging from +0.5 s to -0.5 s. The scan sessions comprised of two experimental runs (10.5 min each), separated by a 6 min high-resolution anatomical scan, and followed by a functional localizer run (7.7 min). Prior to entering the scanner, the participants received training, familiarizing them with the experimental task. The participants indicated the location of the target (i.e., the anomalous quadrant in each array) using response pads, with each quadrant corresponding to a unique button on the response pad. Since the arrays of the Scrambled condition did not contain a target, participants could respond with any button on the response pad in order to advance to the next trial.

### Category Localizer Experiment

**FIGURE 2:**
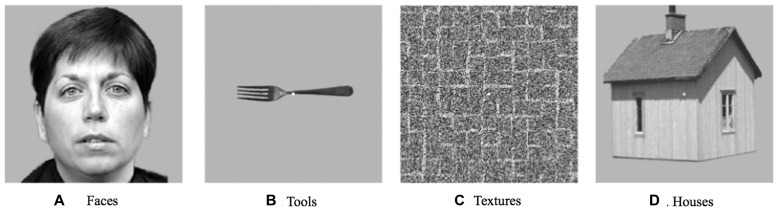
**A subset of the stimuli used in the category localizer experiment. (A)** An example from the faces category. **(B)** An example from the tools category. **(C)** An example from the textures category. **(D)** An example from the houses category.

In addition to the main experiment, an independent functional localizer experiment was conducted in order to identify category-selective regions in visual cortex. This block-designed fMRI experiment included four stimulus conditions (faces, houses, objects, and simple textures; see **Figure 2** for stimulus examples). Each condition was repeated seven times in pseudorandom order. Blocks consisted of 9 images of the same category, each displayed for 800 ms followed by a 200-ms blank screen (a total of 9 s, interleaved with 6-s fixation periods). All stimuli were grayscale photographs of 300 by 300 pixels each, subtending a visual angle roughly equal to that of the experimental arrays. The task was a standard one-back memory task, with an image repetition occurring once or twice in each block.

### Magnetic Resonance Imaging Setup

A 1.5 Tesla MRI scanner (General Electric Excite HDX; General Electric, Milwaukee, WI, USA) with an eight-channel head coil was used for all acquisitions. A high resolution T1-weighted anatomical scan was acquired for each participant using a 3D Magnetization-Prepared Rapid-Acquisition-Gradient-Echo (MPRAGE) sequence (512 × 512 matrix, 120 slices, 1 mm × 1 mm × 1 mm voxel size, TR/TE = 500/15 ms, flip angle = 15°). Three functional MRI acquisitions were also acquired for each participant using a Gradient-Recalled-Echo (GRE) sequence (64 × 64 matrix, 24 slices, 4.5 mm × 4.5 mm × 5 mm voxel size, 1 mm slice gap, TR/TE = 2000/10 ms, and flip angle = 90°).

### fMRI Data Preprocessing

We used the BrainVoyager software package (Brain Innovation, Maastricht, The Netherlands) to analyze the fMRI data. The first image was discarded from each functional scan prior to analysis. Next, the scans were normalized into Talairach space. Preprocessing steps performed on the functional images included 3D motion correction, slice scan time correction, linear trend removal, and mean intensity adjustment.

### Statistical Analysis: Behavior

For all analyses, the 16 levels of configurality were condensed into four Configurality Ranges in order to increase the number of repetitions: Range 1 (0°, 6°, 12°, 18°), Range 2 (24°, 30°, 36°, 42°), Range 3 (48°, 54°, 60°, 66°) and Range 4 (72°, 78°, 84°, 90°). Additionally, a rate of change measure was derived on an individual basis for each participant. A line of best fit was calculated for each participant’s RT data, and the slope of that line was used as our rate of change measure, revealing the rate at which an individual’s RT changes as the configurations depart from parallel and symmetric orientations.

### Statistical Analysis: Neuroimaging

For each subject, after the time courses of the two scans were transformed into Talairach space and preprocessed (see fMRI data preprocessing and analysis), they were z-normalized and concatenated. For the ROI time course analysis, the data were deconvolved using the deconvolution analysis for rapid-event-related paradigms that consists of a general linear model analysis in BrainVoyager software package (Brain Innovation, Maastricht, The Netherlands) in order to extract the estimated hemodynamic response in each voxel for each condition. The analysis was done separately for each subject on a voxel-by-voxel basis. Additionally, rate of change measures were calculated for each participant within each ROI. The rate of change was determined by calculating the slope of each participant’s response magnitude function across the Configurality Ranges, within each ROI. These rates of change measures allow us to quantify how the changes in configuration across Configurality Range reflect changes in the response magnitudes of each individual.

### ROI Selection and Analysis

Regions of interests were identified in each subject separately based on the category localizer experiment as described above. They were defined on the basis of a minimum cluster size of 6 contiguous functional voxels that exhibited selective activations in response to a specific category (*p* < 0.01). EVC ROIs were defined as those contiguous voxels that responded preferentially to textures relative to faces and objects. LOC ROIs were defined as those contiguous voxels that responded preferentially to objects as compared to textures. FFA ROIs were defined as those regions showing preferential activation for faces relative to houses. PPA ROIs were defined as those regions showing preferential activation for houses relative to faces and textures. We applied a deconvolution analysis using the BrainVoyager software package (Brain Innovation, Maastricht, The Netherlands) to the time course of each voxel within the ROIs to extract an estimated hemodynamic response. Next, the estimated responses were averaged across five time points to include stimulus onset, the two points preceding onset, and the two points following onset. This averaging approach, focusing on mean amplitudes rather than peak activations, was chosen to avoid the issues of inter-trial variability, inter-individual variability and inter-trial latency jitters to which peak measures can be susceptible (Luck, 2014). These estimated response values were then averaged across hemisphere, as no significant differences were found between the ROI time courses for the left and right hemispheres, and averaged across participants. These values were then used in an analysis of variance (ANOVA). ANOVAs were also conducted using the peak activation of the estimated hemodynamic responses for each condition, but while descriptively in the same direction, these analyses yielded no significant results.

## Results

### Behavior

To examine the effect of configurality on behavioral performance, we conducted a within subjects repeated measures ANOVA with Configurality Range (five levels: Range 1- Range 4, scrambled) and Run (two levels) as independent variables on participants’ mean response times. Trials in which participants provided inaccurate responses (2.37% of Range 1 trials, 1.41% of Range 2 trials, 2.39% of Range 3 trials and 2.63% of Range 4 trials), and trials identified as outliers (15 out of 1900 trials, <0.01%) were removed from the dataset. Response times were averaged across both location (quadrant) and target direction (facing left or right) as initial analyses did not reveal significant effects of these factors [*F*(3,21) < 1 and *F*(1,7) = 3.04, MSE = 0.370, *p* > 0.12, respectively].

**FIGURE 3:**
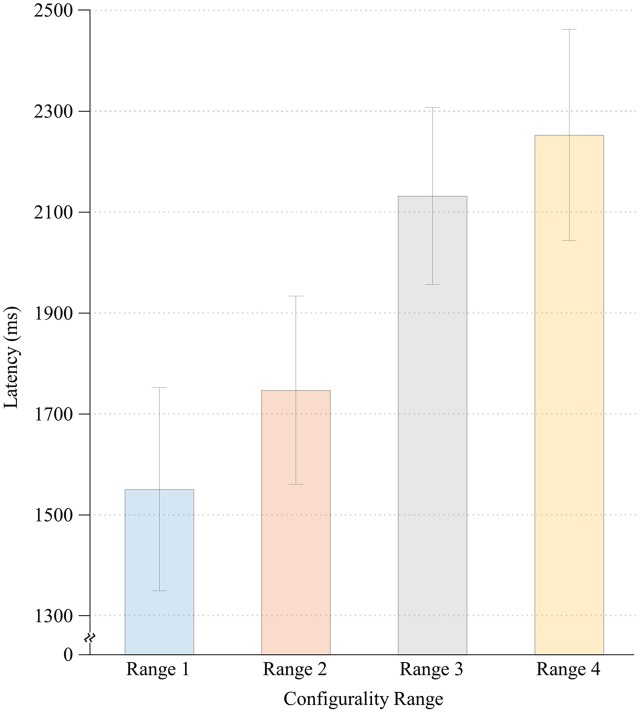
**Mean response times for each Configurality Range with error bars denoting standard error**.

Significant main effects were found for both Configurality Range [*F*(4,28) = 32.36, MSE = 0.17, *p* < 0.001] and Run [*F*(1,7) = 41.82, MSE = 0.012, *p* < 0.001]. Participants responded faster in the second run (*M* = 1.61, *SE* = 0.11) relative to the first run (*M* = 1.77, *SE* = 0.11). However, there was no significant interaction of Run and Configurality Range [*F*(4,28) = 1.30, MSE = 0.008, *p* < 0.30], implying that the differences between the experimental conditions were maintained over time. Consequently, response latencies were averaged across runs for subsequent comparisons. Mean response times are presented in **Figure 3**.

To further explore the effect of Configurality Range, we performed a series of planned comparisons on each successive pairs of our conditions. We found that in general, the smaller the deviation from configurality the faster the response latencies were. Thus, response latencies in Range 1 were significantly lower than those in Range 2 [*F*(1,7) = 14.27, MSE = 0.022, *p* < 0.007], and response latencies in Range 2 were significantly lower than those in Range 3 [*F*(1,7) = 12.12, MSE = 0.231, *p* < 0.01]. Response latencies in Range 3 were faster than in Range 4, although this effect was marginally significant [*F*(1,7) = 4.72, MSE = 0.02,4, *p* < 0.07]. Finally, the response latencies in the Scrambled condition were significantly faster than in each of the four Configurality Ranges (all *p*’s < 0.003).

### Neuroimaging

**FIGURE 4:**
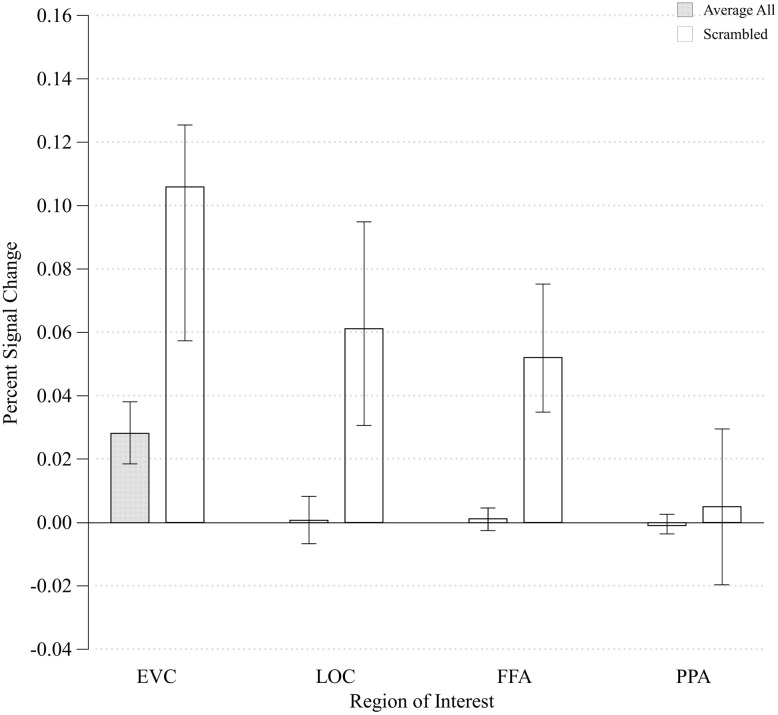
**Mean percent signal change of all Configurality Ranges compared to the percent signal change of the Scrambled condition within each ROI.** Error bars denote standard error.

In order to determine how early along the ventral visual pathway configurality emerges, we examined how our parametrical manipulation of the relations between visual features impacted the magnitude of activation in EVC and higher-level visual areas (LOC). We started by asking whether the configural stimuli would produce differential activation relative to any type of general visual stimulation by comparing the differences in activation between the configural stimuli (averaged across all levels of configurality) and arrays of task-irrelevant, scrambled elements. We conducted a two-way ANOVA with ROI (EVC/LOC) and Stimulus Type (Configural/Scrambled) as our independent variables (no significant effects were found for Run, Hemisphere or their interactions). We found significant main effects of ROI and Stimulus Type [*F*(1,7) = 9.23, MSE = 0.001, *p* < 0.02; *F*(1,7) = 9.65, MSE = 0.005, *p* < 0.02, respectively], and critically, also an interaction between ROI and Stimulus Type, *F*(1,21) = 8.92, MSE < 0.00005, *p* < 0.02). *Post hoc* analyses revealed that the difference between configural and scrambled stimuli was more pronounced in EVC [*t*(7) = -3.58, *p* < 0.01] than in LOC [*t*(7) = -2.38, *p* < 0.05]. However, and somewhat counter-intuitively, the Scrambled condition elicited a higher response than the configural stimuli across ROIs (**Figure 4**). This may be accounted for by the fact that while the scrambled and experimental stimuli were originally designed to have identical physical size (in terms of pixel count; for examples of stimuli, see **Figure 1**), it might be that the density and distribution of these pixels differ across the different types of stimuli, leading to this unexpected effect. This interpretation was supported by an image analysis using V1-like Gabor jet-filter model (Yue et al., 2012; Margalit et al., 2016, see Supplementary Information).

We next examined the extent to which finer differences within the configural stimuli are manifest in the response magnitude of LOC and EVC. We conducted a three-way ANOVA in each ROI with Run (first run/second run), Hemisphere (right/left) and Configurality Range (Range 1- Range 4) as independent variables. We excluded the Scrambled condition from the analysis given its noticeable difference in response magnitude from the other configural stimuli.

### Lateral Occipital Complex

**FIGURE 5:**
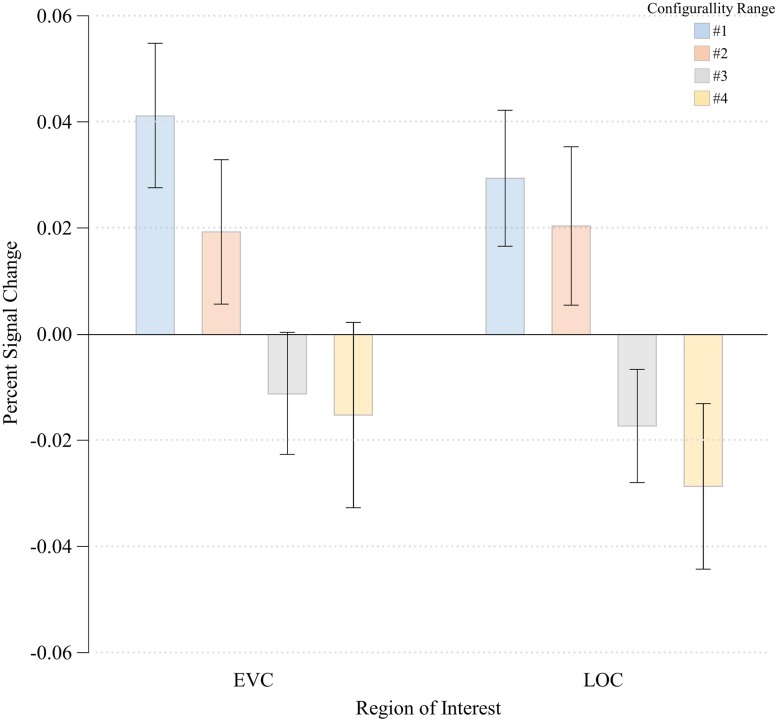
**Percent signal change at each Configurality Range within EVC and LOC.** Error bars denote standard error.

The three-way ANOVA showed no main effects of either Run or Hemisphere [*F*(1,3) = 2.08, MSE = 0.00, *p* > 0.24; *F*(1,3) < 1.00, respectively]. No significant interactions between any of the independent variables were noted (all *p*’s > 0.22). We therefore we averaged our data across these two factors, and performed a one-way ANOVA with Configurality Range as the independent variable. This ANOVA revealed a significant main effect of Configurality Range [*F*(3,21) = 5.33, MSE = 0.001, *p* < 0.007]. It is important to note that given the small sample size, these results should be considered with caution. However, a *post hoc* power analysis revealed an observed power of 0.88. *Post hoc* comparisons of each consecutive pair of configurality ranges, revealed a significant difference in response magnitude between Range 2 and Range 3 [*t*(7) = 1.913, *p* < 0.05], with no significant differences between the upper two and the lower two levels of configurality (all *p*s > 0.45) (**Figure 5**). A linear trend analysis (Keppel, 1991) across all levels of configurality revealed a significant linear trend [*F*(1,7) = 9.71, *p* < 0.02].

### Early Visual Cortex

The three-way ANOVA showed no main effects of either Run or Hemisphere [*F*(1,5) < 1.00; *F*(1,5) < 1.00, respectively]. No significant interactions between any of the independent variables were noted (all *p*’s > 0.12). We therefore averaged our data across these two factors, and performed a one-way ANOVA with Configurality Range as the independent variable. A significant main effect of Configurality Range [*F*(3,21) = 3.36, MSE = 0.002, *p* < 0.04] was obtained. Despite the small sample size, a *post hoc* power analysis yielded an observed power of 0.68. *Post hoc* pairwise comparisons following the main effect of Configurality Range showed that Range 1 stimuli evoked a significantly greater response than Range 2 [*t*(7) = 2.383, *p* < 0.05]. There were no other significant differences between each two successive levels of configurality (all *p* > 0.23). A linear trend analysis across the four levels of configurality revealed a marginally significant linear trend [*F*(1,7) = 4.37, *p* < 0.08]. Thus, EVC also showed an effect of configurality, albeit in a different manner than LOC, as its response separated the highest level of configurality from the other levels of configurality.

To evaluate the possibility that the observed effects of configurality on response magnitudes in EVC reflect differences in the physical image-based properties of the stimuli, we employed the above V1-like Gabor jet-filter model to calculate the model’s predictions of response magnitude in V1 in response to each of the four conditions. We found that the model’s response was roughly equivalent across the first three conditions, with a higher response to Range 4 (see Supplementary Information, Supplementary Figure S4). This pattern of response stands in contrast to the actual responses observed in EVC (as well as LOC), suggesting that the configurality effects in EVC cannot be explained by differences in physical stimulus properties. Nonetheless, it should be noted that a different approach to modeling V1 could potentially support the alternative explanation that low-level stimulus differences produce the observed results in lower level visual areas.

### Other Regions of Interest

**FIGURE 6:**
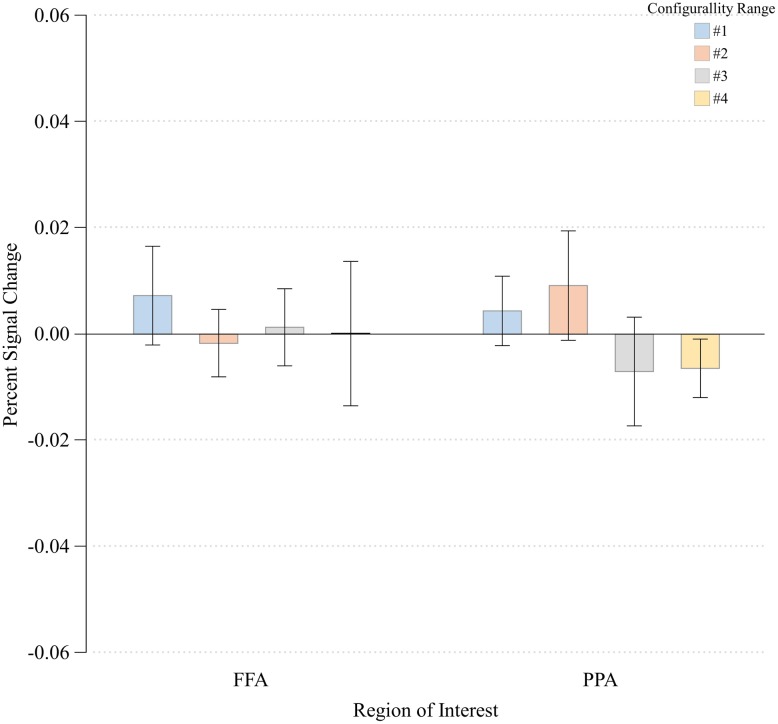
**Percent signal change at each Configurality Range within FFA and PPA.** Error bars denote standard error.

To verify that the main effect of configurality was due to our experimental manipulation rather than reflecting a general effect, such as global attention, we examined the extent to which configurality modulates the magnitude of response of two additional high-level visual regions: the parahippocampal place area (PPA) and the fusiform face area (FFA). We wanted to verify that our findings are not simply due to the fact that participants were more engaged, or more attentive to the most configural stimuli. Given that in addition to their category selectivity, FFA and PPA are susceptible to manipulation of selective attention (O’Craven et al., 1999), we should expect to find a general increase in their response magnitude if indeed our participants were more engaged with the configural stimuli. We first found that activation in response to the Scrambled condition was significantly higher relative to the Configural condition in the FFA [*t*(7) = -3.08, *p* < 0.02]. Response magnitude in PPA was not sensitive to the difference between the two conditions [*t*(7) = -0.31, *p* > 0.75]. We than assessed the effects of Configurality Range on both ROIs (excluding Scrambled, and averaging across runs and hemispheres) by conducting a one-way ANOVA with Configurality Range as an independent factor for each ROI (**Figure 6**). Critically, neither FFA nor PPA showed a significant effect of our conditions [*F*(3,21) < 1.00 for both ROIs], making the possibility of general attentional effect unlikely.

### Correlations With Behavior

To directly assess how the neural measures of configurality reported above are related to the behavioral performance measures, we correlated the rate of change in reaction times as a function of configurality range with the rate of change in response magnitude as a function of configurality range (calculating the slope of each variable for each individual; see Materials and Methods for details). We found a significant negative correlation between the rate of change in reaction times and the rate of change in response magnitude in both EVC (*r* = -0.80; *p* < 0.02) and LOC (*r* = -0.81; *p* < 0.01), indicating that the change in the speed of response to the varying degrees of configurality is tightly linked to the increase in magnitude as a function of configurality. In contrast, FFA and PPA showed no significant correlation between the behavioral change measure and the neural change measure (FFA: *r* = -0.34, *p* < 0.40; PPA: *r* = 0.36, *p* < 0.37).

## Discussion

Emergent features are perceptually salient properties that arise from interactions among local stimulus elements but are not visible within any one element alone (Pomerantz and Portillo, 2011). The goal of the present study was to examine how flexible emergent features are, that is, to determine the extent to which the perception of emergent features is tolerant to changes in the arrangement of their constituent local elements. We manipulated the arrangement of local elements within stimulus arrays, and assessed the extent to which the CSE, an index of stimulus configurality (Pomerantz and Cragin, 2015), can be obtained across a range of deviations from an ideal configuration. We used behavioral performance to establish whether increasing configurality produces faster and more accurate responses; we used neuroimaging to determine where increasing configurality produces increased activation along the ventral visual pathway.

Behaviorally, we found a clear relationship between response times and degree of stimulus configurality. The closer the stimulus was to the ideal configuration, the faster the response latencies were. Neurally, we found effects of stimulus configurality on response magnitude in two key visual areas (EVC and LOC). Further, the effect of configural information was already evident in early stages of processing in the visual hierarchy. While not in total agreement, both our behavioral and neural findings suggest that the CSE is manifest even when the stimuli do not adhere to what is usually considered a “prototypical” configuration. The behavioral findings suggest that the CSE is robust to variations in stimulus configurality, implying that configurality does not operate in an all or none fashion. And while the neuroimaging data are admittedly more equivocal, at the minimum they show that there is a distinction between various types of configurations as a function of how much they deviate from the prototypical configuration, namely, separating “strong” configurations and “weak” configurations.

We hypothesized that configurality is not an all or none phenomenon, and therefore, that variations in symmetry and parallelism would impact the strength of the CSE. In line with our predictions, we found that behaviorally, the CSE weakened as the stimuli departed from what is usually considered to be the optimal configuration (Range 1 stimuli). This decrease was evident until Range 3, after which further deviations no longer resulted in additional decrements to performance. This monotonic relationship between performance and stimulus organization demonstrates that configurality effects can be achieved for stimuli other than the prototypical configural stimuli (e.g., perfectly symmetrical stimuli) and are perceived in a more continuous fashion. In other words, emergent features are still useful for improving task performance even in “suboptimal” forms, that is, in contexts that do not entirely satisfy the geometric properties that usually characterize emergent features. Importantly, we were able to establish when “suboptimal” becomes “non-optimal,” reflected in the plateauing of the RT function at the third and fourth Range levels.

The current study is not the first to suggest that traditional Gestalt properties possess a more continuous nature. For example, Pomerantz and Schwaitzberg (1975) sought to discover the limits of proximity on configural grouping by gradually increasing the separation of individual elements in stimulus arrays. They found that performance declined monotonically as the stimulus elements were more widely spaced, although this effect of proximity was eventually restricted in range (<4° of visual angle). Our findings are similar to those of Pomerantz and Schwaitzberg (1975) who also found a similar monotonic relationship, as well as an established point at which no further advantages were conferred by the stimulus configural structure. Moreover, the current study extends Pomerantz and Schwaitzberg’s (1975) findings to additional stimulus properties other than proximity, namely, parallelism and bilateral symmetry. More generally, one implication of the current findings is that other seemingly binary principles of object perception may prove to be more flexible than they are often considered. One potential example might be relatability, a criterion used to solve the grouping problem (Kellman and Shipley, 1991; for a review see van Lier and Gerbino, 2015). This criterion predicts a-modal completions by a smooth curve when linear extensions would meet behind the occluding surface at angles of 90° or larger, but not at smaller angles (van Lier and Gerbino, 2015) While this implies a binary rule, an outstanding question would be to what extent the percept of completion is affected by a continuous manipulation of the angle as it progresses farther away from 90°.

It should be noted that the CSE is traditionally defined based on a comparison between display elements in isolation and the same display elements within a context, and thus, technically speaking, we have not measured the CSE here. However, the chosen stimuli of parenthesis pairs we have used in the current study have been repeatedly shown to elicit strong CSEs when contrasted with isolated parentheses (Pomerantz and Garner, 1973; Pomerantz et al., 1977; Mersch, 2014), thereby allowing us to exclude an “isolated” condition, and focus instead on the range of configural variations.

Pomerantz (1986, p. 8) once observed that the perception of emergent features is “dependent on the identity and arrangement of the parts.” This has some practical real world implications, particularly for the design of dynamic graphical displays (i.e., those that appear on the interface of a sociotechnical system), as compared to static graphical displays (i.e., those that appear on the printed pages of a book). The arrangement of parts in dynamic displays will constantly be in flux because they are directly coupled to the changing variables and properties of the work domain. Therefore, different spatial arrangements of exactly the same parts in the display can produce emergent features that are salient in one case but not in another. The upshot is that the meaning behind the displays can be distorted or lost, resulting in dire consequences. One direct implication therefore is that dynamic displays must be developed and evaluated under experimental contexts that allow the range of their dynamic behavior to be observed (e.g., dynamic simulations of the work domain). A second practical consideration is that a better understanding of the neural substrates underlying the perception of emergent features (such as in the present study) can lead to more effective design guidelines and safer systems (i.e., the neuroergonomic approach: Parasuraman and Wilson, 2008).

In line with the latter point, having established the non-dichotomous nature of CSE using behavioral performance, we also asked whether this effect could also be observed at the neural level. We found that configurality plays a significant role in driving the response of both object-selective (‘high’) and early (‘low’) visual cortex, as reflected in a main effect of Configurality Range in LOC and EVC, respectively. However, while configurality effects were found in both EVC and LOC, the pattern of information usage was not uniform across the ROIs. LOC responded with equal magnitude to both Range 1 and Range 2 stimuli, indicating that despite the range of variations, on average, it treats all stimuli within those ranges of configurality as equally configural. In other words, LOC’s response is tolerant to at least some deviation from the ‘prototypical’ configuration (although LOC did not differentiate among the other two ranges). The response of EVC was more selective, sensitive only to the most configural stimuli (Range 1), as its response was highest to that condition and could not further distinguish among the other configurations. Further resonating this pattern of quantitative rather than qualitative difference between the two regions, we found that response magnitude in LOC scaled linearly with increase in configurality range, while in EVC such a linear trend was also observed, albeit marginal.

These differential patterns of information usage in EVC and LOC might suggest that visual processing of configural information varies across different regions in the ventral visual pathway. One potential interpretation for this functional difference is that the degree of tolerance to deviations from configurality might be determined as a function of location along the visual pathway. Thus, farther upstream, EVC is very specific and responds only to the most configural stimuli (Range 1: 0° – 18° of rotation). In contrast, farther downstream LOC responds equally to all stimuli across Range 1 and Range 2 (0° – 42° of rotation). This putative increase in tolerance to variations in configurality parallels similar increases in tolerance observed with other object dimensions along the ventral visual pathway (for a review, see DiCarlo et al., 2012). Such an account, however, should be taken with caution at this point, as the current neural data are more limited in nature compared with the more robust behavioral findings. Whereas the behavioral data shows a clear monotonic relationship between configurality and performance, the exact pattern of the relationship between configurality and neural activity is less clear in the current dataset.

Interestingly, in spite of the notion of increased representational complexity, the current study shows that one can observe a difference in activity based on the perceptual organization of the features as early as EVC. This is in contrast to prior works showing that EVC (primarily V1) activity does not capture the difference between local stimulus features and the perception of global shape they produce, whereas LOC supports global processes that go beyond the processing of isolated features (Hasson et al., 2001; Lerner et al., 2001). Under this view, EVC would not be anticipated to show any difference in its response across the range of configural stimulus arrays, whereas LOC would. Consistent with this prediction, a recent fMRI study of the CSE (Kubilius et al., 2011), comparing the processing of contextually configured stimuli (‘wholes’) relative to similar stimuli that did not contain configural context (‘parts’) found that LOC showed a higher classification performance for wholes versus parts, whereas EVC (V1, V2, V3) showed an opposite pattern, with lower classification to wholes versus parts. Based on these results, the authors concluded that the CSE manifests in higher visual areas, not in ‘low-level’ visual areas.

While seemingly at odds with the current results, several methodological differences make a direct comparison between the two studies difficult. For instance, the current study used a univariate analysis of the response magnitude, rather than a multi-voxel pattern analysis (see Ostwald et al., 2008 for a discussion of this point). Interestingly, while Kubilius et al. (2011) also report response magnitude data, they did not find any significant difference between the whole and parts conditions, either in EVC or in LOC (Supplemental Material). And, whereas Kubilius contrasted display elements in isolation with the same display elements within a context, we did not use an “isolated” condition, but rather employed a range of context-bound configural variations (see above).

In spite of the methodological differences between the studies, it is important to discuss how the two studies relate to one another from a theoretical perspective. The current study focused on establishing how gradually varying the relationship between stimulus elements impacts visual cortex, rather than contrasting parts and wholes in ‘low’ and ‘high’ level visual cortex. In other words, instead of assuming a clear-cut separation between ‘low-level’ parts and ‘high-level’ wholes, we sought to investigate the nature of the transition between configural “wholes” and non-configural “parts.” Asking how early in the visual processing stream we can find sensitivity to subtle changes in configuration leads us to conclude that configurality (or “wholeness,” to maintain the analogy) is not a binary variable, and critically, that it is supported by the activity of multiple visual regions. Arguably, the joint activity of these visual regions – each differentially representing various aspects of the configural range – culminates in the characterization of the full range of the stimulus array.

The current idea that emergent features are neurally represented by the conjoint activity of multiple regions along the ventral visual pathway is in line with prior neuroimaging works that pointed to the role of both early and higher visual cortex in utilizing emergent features such as collinearity and orientation for the perception of global shapes (Kapadia et al., 1995; Sigman and Gilbert, 2000; Altmann et al., 2003; Kourtzi et al., 2003; Ostwald et al., 2008). These studies mark a departure from the traditional, feedforward theory of cortical organization, and support a top-down interactive view of the ventral visual pathway (Croner and Albright, 1999; Lamme and Roelfsema, 2000; Kravitz et al., 2013). Under this view, the ventral visual pathway is a recurrent and highly interactive occipitotemporal network that bridges EVA and anterior temporal lobe along multiple routes through which visual information is processed. According to one specific conceptualization, this occipitotemporal network is hypothesized to be involved in the processing of ‘stimulus quality’, that is, the formation of specific representations or associations involving stable aspects of visual information (Kravitz et al., 2013).

The term ‘stimulus quality’ is particularly relevant to the current discussion, as this term aims to capture available information at its broadest sense, referring to the processing of both perceptual dimensions and their conjunction. Any stimulus according this view can be represented as “a coordinate or configuration along all of the dimensions that the occipitotemporal network represents” (p. 28). Arguably, the reason for having multiple representations of configurality might stem from the need for flexible object categorization, whereby different visual cues become diagnostic as a function of the specific task or recognition goal of the observer (Schyns, 1998; Harel and Bentin, 2009). Notably, these top–down modulations of visual processing are supported by both intrinsic interaction in the ventral visual pathway and extrinsic interactions between the ventral visual pathway and other a-modal higher-level areas outside of OTC (e.g., Harel et al., 2014; Chiou and Lambon Ralph, 2016). This hypothesis is obviously beyond the scope of the current work, as the current study used only a single task (visual discrimination). However, future studies that will parametrically manipulate both stimulus configurality and task relevance constitute a promising direction of research.

## Author Contributions

OF and KB designed the study; OF conducted the study; OF, AH, and KB analyzed the data; OF, AH, and KB wrote the paper; all authors read and approved the manuscript.

## Conflict of Interest Statement

The authors declare that the research was conducted in the absence of any commercial or financial relationships that could be construed as a potential conflict of interest.
