# Supplementary material for: How Configural Is the Configural Superiority Effect? A Neuroimaging Investigation of Emergent Features in Visual Cortex
Source: Front Psychol. 2017 Jan 23;8:32. doi: 10.3389/fpsyg.2017.00032 (PMC5253576; doi:10.3389/fpsyg.2017.00032)
Supplement: Supplementary file 1 [file Data_Sheet_1.DOCX]

**Supplementary Materials**

**Gabor-Jet Model: Background and Methods**

To evaluate our stimuli for potential low-level differences, we analyzed our stimulus set using a Gabor-jet model of primary visual cortex (V1). The Gabor-jet model (Lades et al., 1993) was created to model the response properties of simple cells in V1 hyper-columns, and computes the psychophysical similarity of images based on V1 simple and complex cell Gabor-like filtering. The model provides a single value per pair of images, for each possible pair, representing the degree of similarity between the two images.

In the Gabor-jet model we implemented, each Gabor “jet” is modeled as a set of Gabor wavelet kernels at 5 scales and 8 orientations, with the center of their receptive fields tuned to an identical location in the visual field. Following the methods of Xu and colleagues (2009), we employ a 10x10 square grid to mark the positions in the image space from which kernel convolution values are extracted, resulting in 100 jets. Next, a 2-D Fourier transform on the image is performed and the Fourier-transformed image is multiplied pixel-wise with the Gabor wavelet kernels. The image is then transformed back out of the frequency domain. The magnitude of the resultant image is extracted at the positions denoted by the 10x10 grid. The magnitudes of each jet are next concatenated, yielding a 100x40 matrix of values for each image, which can then be collapsed into a vector of 4000 values.

We then utilized these 4000 model response magnitude values to run a series of analyses. First, we conducted a simple cross-correlation analysis between every possible pair of images, yielding a correlation matrix that represents image-based similarity as viewed through the perspective of the above-mentioned V1 model. Second, we calculated an average response magnitude for each Configurality Range by averaging the values generated by the model in response to all of the images comprising each Configurality Range.

**Results & Discussion**

**Image Similarity.** An examination of the image similarity matrix of all images used in the experiment (a total of 144 images) provides several key insights into the nature of the stimuli. First, we observed relatively low correlation values between the experimental stimuli (configural arrays; first 128 cells) and the scrambled images (the last 16 cells). This implies that according to this particular V1 model, the two types of stimuli are very distinctive (i.e. they are dissimilar to one another: Figure S1). In fact, in spite of the range of variations in orientation, quadrant, and target identity (either defined by parallelism or symmetry), the entire group of experimental stimuli is still grouped together relative to the scrambled stimuli, suggesting a qualitative difference between the experimental and scrambled stimuli. This qualitative difference can be summarized by one common characteristic of the experimental stimulus set, namely, their configural nature.

Next, we considered the experimental stimuli in isolation, averaging across the individual variants of each degree of rotation (see Methods in main text). This analysis reveals a pattern of image similarity that is gradual and continuous in nature (Figure S2). Specifically, there is an apparent decrease in similarity with increasing distance from the “optimal” configuration. This continuous pattern of image similarity serves as another evidence for the parametric nature of the current stimulus set.

Lastly, the model was able to approximate the detailed structure of our stimulus set. Within each of the 16 degrees of rotation, there were 8 individual stimuli that varied in target location (upper left, upper right, lower left, lower right) and target identity (defined either by parallelism or symmetry: see main text for details). Examining the similarity matrix (Figure S1), one can observe various similarity patterns that directly reflect these variations within a given degree of rotation (i.e. every eight cells). Both the target’s identity (i.e., the emergent feature) and the target’s location are discriminable. Interestingly, stronger similarities are seen when the target identity matches than when the target location matches, and further, one can observe groupings that differentiate both the left and right hemi-fields as well as the upper and lower visual fields (regardless of the target’s identity). Specifically, the highest correlations (except for the diagonal) were observed between the same target when appearing in the same hemi-field across variations in the upper and lower visual field. In contrast, the lowest correlations were observed when moving across the hemi-field and target identity. For ease of visualization, we have “zoomed in” on the eight variants within the first range of configurality (Figure S3). Note that this pattern is consistent across all ranges (Figure S1).

**Model Response Magnitude.** In order to more closely relate the output of the Gabor-Jet model with the analyses performed on the actual response magnitude data observed in EVC, we calculated an average model response magnitude for each of our four Configurality Ranges. If the observed effects of configurality on response magnitude in EVC were attributable to physical, image-based properties of the stimuli, we would expect the model response magnitudes to differ across our Configurality Ranges. What we see, however, is that the model response magnitudes are roughly equivalent across the four ranges of configurality (Figure S4). Notably, the pattern of the estimated V1 responses generated by the model is not consistent with the pattern of the actual response magnitudes observed in EVC. This suggests that the configurality effects observed in EVC are likely not due to physical image properties, at least not as approximated by the Gabor-jet model.

*
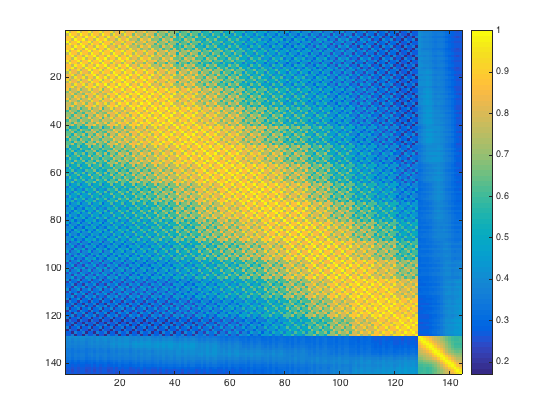
*

*Figure S1.* The V1 Gabor-jet model similarity matrix quantifying the similarities among the stimulus set, including both the experimental and Scrambled stimuli. The experimental stimuli are presented first, in order of the Configurality Ranges, followed by the 16 Scrambled stimuli (color scale represents Pearson’s correlation values).


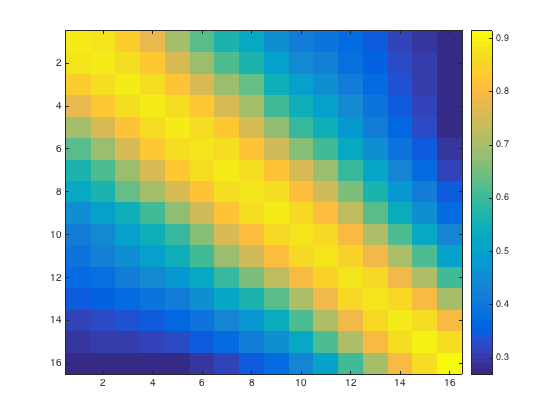
*Figure S2.* The V1 Gabor-jet model similarity matrix quantifying the similarities among the stimulus set, excluding the Scrambled stimuli and averaged across the 8 variations of each rotational range. A clear, continuous pattern of similarity is observed.

*
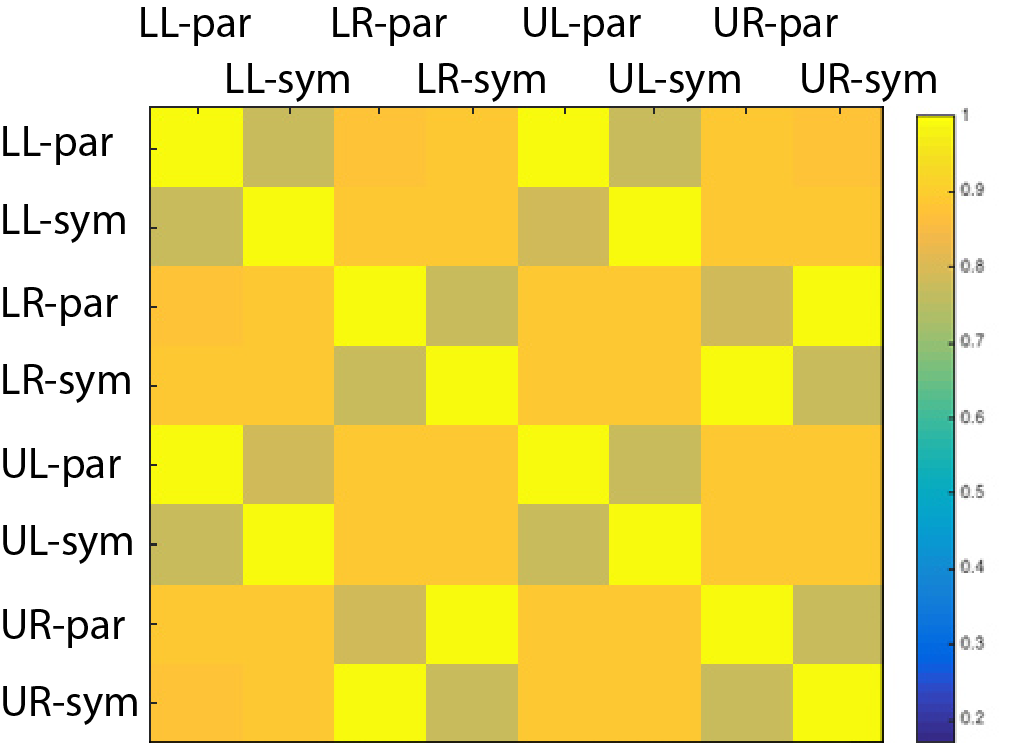
*

*Figure S3.* A more detailed view of the similarity matrix representing the 8 variations of stimuli within the first degree of rotation. This matrix shows the relationships among the variations in the quadrant location of the target (lower left (LL), lower right (LR), upper left (UL), and upper right (UR)) and variations in the targets identity (defined by either parallelism (par) or symmetry (sym)).


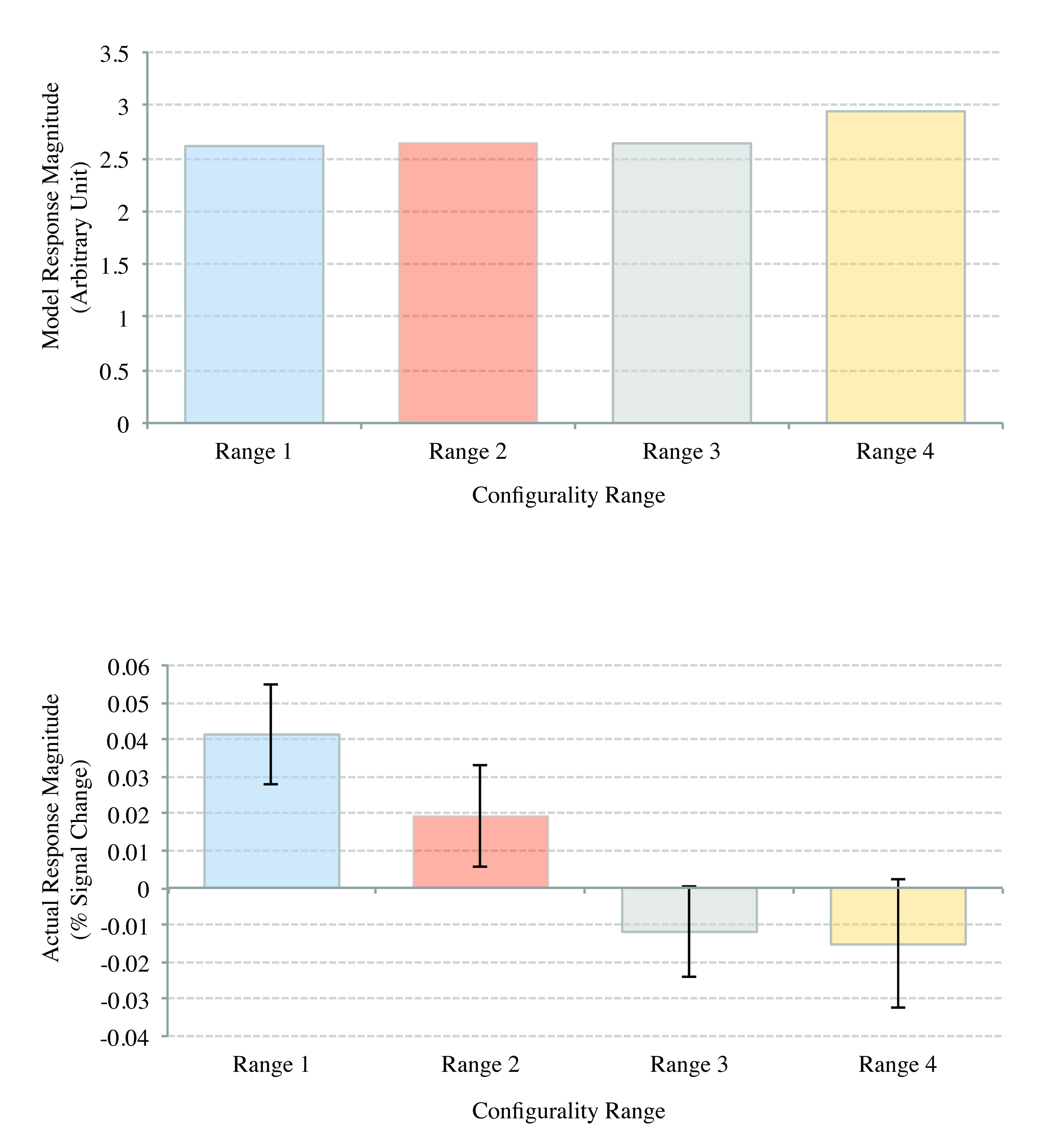


*Figure S4.*Top: Average Gabor-Jet model response magnitude for each Configurality Range is shown at the top. Bottom: Actual response magnitudes observed in EVC (taken from Figure 5 of the main text).

**References**

Lades, M., Vorbruggen, J.C., Buhmann, J., Lange, J., von der Malsburg, C., Wurtz, R.P., & Kongen, W. (1993). Distortion invariant object recognition in the dynamic link architecture. IEEE Transactions on Computers, *42*, 300-311. doi: 10.1109/12.210173

Xu, X., Yue, X., Lescroart, M.D., Biederman, I., & Kim, J.G. (2009). Adaptation in the fusiform face area (FFA): image or person?. *Vision research, 49*, 2800-2807.
